# Loss of E-cadherin promotes prostate cancer metastasis via upregulation of metastasis-associated gene 1 expression

**DOI:** 10.3892/ol.2020.11421

**Published:** 2020-02-28

**Authors:** Liangsheng Fan, Hongyan Wang, Xi Xia, Yumei Rao, Xiangyi Ma, Ding Ma, Peng Wu, Gang Chen

Oncol Lett 4: 1225-1233, 2012; DOI: 10.3892/ol.2012.934

Subsequently to the publication of the above paper, the authors were alerted to the fact that some of the experimental images featured in Fig. 3C had been selected inappropriately. Specifically, the images presented for the ‘Mock’ and ‘Control siRNA’ experiments had appeared in another article published by the same authors. These errors occurred during the preparation of the manuscript, and were attributed to the inadvertent mislabelling of images or folders.

The corrected version of Fig. 3, showing the correct data for the ‘Mock’ and ‘Control siRNA’ experiments, is shown opposite. Note that these errors in the data selection for this figure did not seriously affect the overall conclusions reported in the study. The authors are grateful to the Editor for granting them the opportunity to publish this Corrigendum, and apologize to the readership of the Journal for any inconvenience caused.

## Figures and Tables

**Figure 3.**
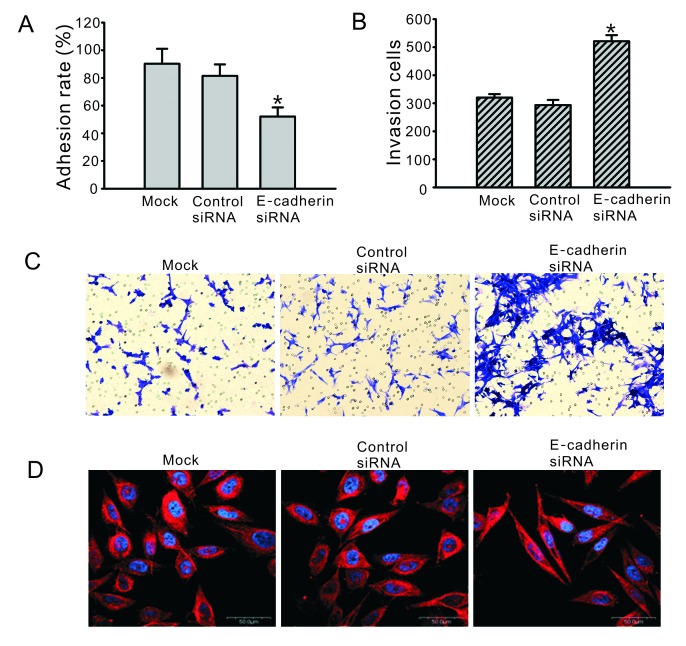
Downregulation of E-cadherin affects cell adhesion, invasion ability and cytoskeleton construction. Cells were transfected with E-cadherin siRNA or negative control siRNA for 48 h, and the adhesive ability was examined by MTT assay. All experiments were repeated three times. (A) The adhesion ability to solid phase was significantly downregulated in cells treated with E-cadherin siRNA. *P<0.05 compared with non-treated cells (mock) and negative control siRNA transfected cells. (B) Invasion ability was assessed using the Matrigel™-coated transwell system. 8×10^3^ cells/well were placed in a Matrigel-coated Boyden chamber and allowed to invade for 24 h. Graph reveals the quantification of invasive cells from the lower side of the transwell inserts. *P<0.05 compared with non-treated cells (mock) and negative control siRNA transfected cells. (C) Representative images of invasion experiments. (D) The altered structure (600-fold) of the cytoskeleton was detected using confocal microscopy. Red fluorescent staining represents the α-tubulin, and blue staining represents the nucleus. siRNA, small interfering RNA; MTT, 3-(4,5-dimethylthiazol-2-yl)-2,5-diphenyltetrazolium bromide

